# Selenium, Selenoproteins, and Female Reproduction: A Review

**DOI:** 10.3390/molecules23123053

**Published:** 2018-11-22

**Authors:** Izhar Hyder Qazi, Christiana Angel, Haoxuan Yang, Bo Pan, Evangelos Zoidis, Chang-Jun Zeng, Hongbing Han, Guang-Bin Zhou

**Affiliations:** 1 Farm Animal Genetic Resources Exploration and Innovation Key Laboratory of Sichuan Province, College of Animal Science and Technology, Sichuan Agricultural University, Chengdu 611130, China; vetdr_izhar@yahoo.com (I.H.Q.); yanghaoxuan940712@gmail.com (H.Y.); bopan1992@163.com (B.P.); zengchj@sicau.edu.cn (C.-J.Z.); 2 Department of Veterinary Anatomy & Histology, Shaheed Benazir Bhutto University of Veterinary and Animal Sciences, Sakrand 67210, Sindh, Pakistan; 3 Department of Veterinary Parasitology, College of Veterinary Medicine, Sichuan Agricultural University, Chengdu 611130, China; qazi5502@yahoo.com; 4 Department of Veterinary Parasitology, Faculty of Veterinary Sciences, Shaheed Benazir Bhutto University of Veterinary and Animal Sciences, Sakrand 67210, Sindh, Pakistan; 5 Department of Nutritional Physiology and Feeding, Faculty of Animal Science and Aquaculture, Agricultural University of Athens, 75 Iera Odos, 11855 Athens, Greece; ezoidis@aua.gr; 6 National Engineering Laboratory for Animal Breeding, Key Laboratory of Animal Genetics and Breeding of the Ministry of Agriculture, Beijing Key Laboratory for Animal Genetic Improvement, College of Animal Science and Technology, China Agricultural University, Beijing 100193, China; hanhongbing@cau.edu.cn

**Keywords:** female reproduction, fertility, GPX1, infertility, ovarian function, oxidative stress, pre-eclampsia, selenium, selenoproteins

## Abstract

Selenium (Se) is an essential micronutrient that has several important functions in animal and human health. The biological functions of Se are carried out by selenoproteins (encoded by twenty-five genes in human and twenty-four in mice), which are reportedly present in all three domains of life. As a component of selenoproteins, Se has structural and enzymatic functions; in the latter context it is best recognized for its catalytic and antioxidant activities. In this review, we highlight the biological functions of Se and selenoproteins followed by an elaborated review of the relationship between Se and female reproductive function. Data pertaining to Se status and female fertility and reproduction are sparse, with most such studies focusing on the role of Se in pregnancy. Only recently has some light been shed on its potential role in ovarian physiology. The exact underlying molecular and biochemical mechanisms through which Se or selenoproteins modulate female reproduction are largely unknown; their role in human pregnancy and related complications is not yet sufficiently understood. Properly powered, randomized, controlled trials (intervention vs. control) in populations of relatively low Se status will be essential to clarify their role. In the meantime, studies elucidating the potential effect of Se supplementation and selenoproteins (i.e., GPX1, SELENOP, and SELENOS) in ovarian function and overall female reproductive efficiency would be of great value.

## 1. Introduction

Selenium (Se) is known as an essential trace mineral that has several important functions at the level of the cell and organism in animal and human health, and therefore it is relevant to various patho-physiological conditions [1]. The biological functions of Se are primarily carried out by selenoproteins (selenium-containing proteins, products of twenty-five genes in human and twenty-four in mouse), which are reportedly demonstrated in all three realms of life, viz. eukaryotes, archaea, and eubacteria, and they were also observed in viruses [2,3,4]. As a component of selenoproteins, Se plays structural and enzymatic roles; in the latter context it is best recognized for its catalytic and antioxidant activities [1].

Among identified mammalian selenoproteins, only a few have hitherto been functionally characterized [4]. Albeit selenoproteins represent various molecular pathways and perform a variety of essential biological functions; they all contain at least one selenocysteine (Sec), a Se containing amino acid (The 21st naturally occurring “proteinogenic” amino acid, as it is inserted into proteins during biosynthesis, in contrast to post-translationally modified amino acids [3,5]), and the majority of them perform oxido-reductase roles [4,6]. Important cellular processes, including synthesis of desoxynucleotides for deoxyribonucleic acid (DNA), scavenging of detrimental signaling peroxides, reduction of oxidized proteins and membranes, redox signaling control, metabolism of thyroid hormones, protein folding and Se transportation and storage, are thus far reported to depend on selenoproteins [4]. The brief descriptions of select mammalian selenoproteins having relevance to fertility, reproduction, and development are summarized in Table 1.

Selenium is generally supplemented as organo-Se compounds or in the form of inorganic Se. It has been demonstrated that the form of Se has an important bearing on its potential beneficial and/or untoward toxic effects on the health and growth of organism [7,40,41]. A consideration of Se speciation in plant and animal food origins is of great importance and requires a thorough apprehension of the putative bio-synthetic mechanisms through which Se is assimilated in plants and how such forms undergo metabolism in animals [42]. A rising discernment is apparent regarding the fact that both total uptake and the form of dietary Se consumed are of equal importance in terms of the health benefits produced. Nevertheless, the latter may be of greater value in this regard [42]. The recommended dietary allowance (RDA) of Se in different mammalian species is listed in Table 2.

Few studies on mammalian species have demonstrated the association between Se status and reproductive function in both males [52,53] and females [53,54]. There is evidence regarding the implication of Se in a number of adverse pregnancy health conditions such as pre-eclampsia, autoimmune thyroid disease, miscarriage, and preterm birth [29]. Moreover, in cattle, Se deficiency might have significant bearing and an economic impact on aspects such as decreased fertility rate, retention of placenta, and the occurrence of reproductive diseases; i.e., metritis [55]. The increased fertility following the addition of Se is ascribed to the decrease in embryonic death during the first thirty days of pregnancy [55]. Most female based studies are mainly centered on the role of Se in pregnancy [56,57,58,59,60]; only recently has some light been shed on its potential role in oocyte development and ovarian physiology [8,53,61].

## 2. Transport of Se and Selenoproteins

It has been reported that Se inadequacy causes a fall in the concentration of selenoproteins [62] Bösl and colleagues demonstrated that the inability of the homozygous *Trsp* (−/−) embryos to synthesize selenoproteins contributes to their embryonic death [63]. Selenoprotein synthesis is mediated by tRNA^Sec^; however, it is unlikely that the amount of tRNA^Sec^ itself is the rate-limiting component in selenoprotein synthesis. It is rather likely that the availability of Se itself plays an essential role [63]. Thus, it is necessary for the pregnant dam to supply the embryo and the fetus with Se to support selenoprotein synthesis [14]. The placental transfer of Se is bidirectional, and might have potential bearing on its net retention in tissues of dam, fetus, and neonate [7,64]. The phenomena that, whether Se readily crosses the placental tissues in women or is, in reality, saturated and contained in the placental tissues, remains largely elusive [7,65]. Two extracellular selenoproteins, viz. selenoprotein P (Selenop) and glutathione peroxidase 3 (Gpx3), contain ca. 97% of Se in mouse plasma [14,66]. Selenoprotein P, produced largely in liver, carries Se to most of the other tissues [14,67], however, information regarding the involvement of Gpx3 in Se transport is relatively sparse [14]. As for Selenop, its transport is mediated via binding with Apolipoprotein E receptor-2 (apoER2), which serves as a mediator for the endocytosis of Se from systemic circulation [14]. Placental apoER2 seems to possess Selenop intake attributes similar to apoER2 in many other organs and tissues [14]. Lately, it has been reported that Selenop and Gpx3 are potentially involved in the transport of Se from the pregnant female to the developing fetal tissues and organs [14]. These findings lend exciting insights regarding the putative mechanisms (yolk sac and placental mechanisms) by which the transfer is achieved [14].

In a mouse model, it has been demonstrated that the visceral yolk sac mediated maternal-fetal Se transfer occurs in early pregnancy and later in gestation via the placenta [14,68]. The visceral yolk sac absorbs the fluid from the uterus that includes Selenop and Gpx3, whereas the placenta imbibes Selenop from dam’s blood through apoER2-arbitrated endocytosis [14]. These two uptake mechanisms are selenoprotein dependent and work during bodily circumstances such as Se inadequacy [14]. Nevertheless, there is a precedent regarding Se transport that does not seem to be dependent on Selenop and Gpx3 (plasma selenoproteins) [14]. Mice with both *Selenop* and *Gpx3* knocked out were viable when given a high Se diet, indicating the implication of other routes by which Se could reach tissues [14,66,68]. Therefore, there seems to be two levels of specific Se transfer; i.e., the lower-tier mechanism and the upper-tier mechanism [14]. The lower-tier mechanism (possibly involving selenosugar or another small-molecule form of the element) does not seem to have direct dependence on selenoproteins [14]. However, it is highly dependent on the Se status of the pregnant dam and, thus, gets less efficient in conditions of Se inadequacy and functions under Se-adequate conditions [14,68]. On the other hand, the upper-tier mechanism (Selenop-apoER2 mechanism [69]) ensures the transport of Se to target tissues according to their need, even when dietary Se is limiting [14]. As demonstrated by Burk et al., the Se transport via the visceral yolk sac has the features of an upper-tier transport mechanism [14]. Even though it is not mediated via selenoproteins receptors, it obviously depends on selenoprotein intake by cells and seems to function in conditions of Se inadequacy [14]. Therefore, both the visceral yolk sac and the placenta function through the upper-tier mechanism (selenoprotein-dependent) for maternal-fetal Se transport that should protect the fetus in Se-deficient pregnant females [14,68].

## 3. Expression of Selenoproteins in Female Reproductive Tissues

Both the inorganic and organic forms of Se are reportedly involved in regulation of the expression of SELENOP [7]. It has been demonstrated that the content of Se and the expression of selenoproteins concurrently rise in placenta during the gestation period in rats [7]. Many selenoproteins, viz. Selenop, iodothyronine deiodinase 3 (Dio3) and thioredoxin reductase (Txnrd), are expressed in the uterus [7,70]. Meanwhile, evidence is also available regarding the expression of other selenoprotein genes such as *GPX1*, *GPX2*, *GPX3*, *GPX4*, *SELENOS*, (also called *VIMP*), *SELENOT*, *SELENOF*, *SELENOH*, *SEPHS2*, *DIO3*, *DIO1*, and *SELENOM* in granulosa cells of bovine ovaries [8]. Up-regulated expression of *SELENOP* and down-regulated expression of *GPX1* and *GPX3* have been reported in small atretic bovine follicles with respect to healthy bovine follicles [71]. Altered placental and uterine Selenop expressions have been observed with advancing pregnancy in mice [7,72]. Usually the level of Selenop expression increases four days prior to the birth and reaches its maximum level at full-term in mice [72]. However, preterm expression of Selenop is also witnessed in fetal liver in mice [72].

Thyroid hormones have significant importance in mammalian reproduction efficiency and during developmental stages [73]. Insufficient production of thyroid hormones in humans and rodents has been linked to reduced fertility, disturbances in the estrus cycle, impaired architecture of the uterus and implantation, and other problems relating to the maintenance of gestation [73]. Galton and colleagues reported significantly higher expression levels of Dio3 mRNA in uteri of pregnant rats [30]. The expression of Dio3 is time and region specific; i.e., highly expressed as early as day nine of gestation and localized in mesometrial and anti-mesometrial decidua of the uterus [7,30]. Both Dio3 mRNA and activity were observed at the site of implantation [30]. This high expression level of Dio3 at the implantation site is presumptuously entailed to prevent the developing fetus from excess exposure to maternal thyroid hormones [7,30]. Likewise, in human placental cells, DIO3 activity is raised with increasing gestational age [7,74]. This evidence strongly indicates that the pregnant uterine placental tissues have an essential function in regulating the exposure levels of the fetus to maternal thyroid hormones [30]. Higher DIO3 expression has also been reported in the syncytiotrophoblast and cytotrophoblasts, endothelial linings of vessels in the placenta and amnion sheath in the umbilical cord, deciduas in uterus, epithelium of human fetus, and non-pregnant human endometrium [31]. These findings indicate that the regional modulation of thyroid status is crucial at all phases of reproduction in females [31]. Many other factors are considered to play their role in the placenta and the uterus [7]. The redox proteins thioredoxin (TRX) and TXNRD have location-specific expressions in human and rodent placenta [7,75,76]. Histochemical observations have revealed that both of these are concentrated in trophoblasts, epithelial tissue of the endometrium and stromal cells in the stem villi [7,75,76]. They are reported to protect the tissues of placenta during inflammatory conditions [7,75,76].

## 4. Selenium in Follicular Development and Ovarian Function

Growth of granulosa cells is considered an important feature during the developmental process of follicles; i.e., the folliculogenesis. The proliferation of small primary follicles (with fewer granulosa cells) to maturing pre-ovulatory follicles (with many strata of cells) is the characteristic event in folliculogenesis [77]. In animal experiments, it has been demonstrated that Se might regulate the growth of the granulosa cells and 17β-estradiol bio-synthesis in adult ovaries in vitro [77]. Recent studies have also elucidated that Se and selenoproteins levels are increased in large healthy follicles and might perform a vital antioxidant function during later growth and the proliferation of follicles [8]. However, the regulatory role that Se is believed to play in the function and development of ovaries in a fetus remains poorly understood [78]. Grazul-Bilska and colleagues have reported that high Se levels (80 μg/kg bw) in a maternal diet repressed the growth of primordial, secondary, and antral follicles, stroma, and blood vasculature in ovaries of fetus in sheep compared to the Se adequate group (6 μg/kg bw). The number of proliferating primordial follicles and the labeling index (percentage of proliferating cells) of primordial follicles was reduced following high Se diets [78].

### 4.1. Evidence from In Vitro and Animal Model Studies

Sodium selenite (inorganic Se) not only improves the oocyte growth, but also enhances the proliferation rate in theca and granulosa cells. In corroboration to this hypothesis, an in vitro study conducted by Basini and Tamanini showed that sodium selenite (5 ng/mL) supplementation triggered the proliferation of bovine granulosa cells and provided some stimulatory effects on E_2_ synthesis. These effects could be arbitrated, at least in part, via repression of nitric oxide production [77]. Furthermore, there is also a possibility that these effects may be influenced by the unidentified Se-dependent antioxidant enzymes in granulosa cells [77]. In another in vitro study, Kamada and Ikumo have demonstrated the implication of Se (5 and 200 ppb added in culture medium) in ameliorating the proliferation of bovine luteal cells, potentially by limiting the detrimental effects of ROS [79]. Moreover, it has been reported that GPX simulates the ability of FSH to repress apoptosis in cultured ovarian follicles of rat [80]. Additionally, Se deficiency has been demonstrated to give rise to ovarian degeneration and atresia of follicles in rats [81]. Recently, Yao and colleagues conducted an in vitro study to elucidate the underlying mechanisms through which Se exerts its effects on the proliferation and steroid hormone bio-synthesis in luteinized-granulosa cells in goat [61]. The biomarkers such as proliferating cell nuclear antigen (PCNA), Akt, and phosphoinositide 3-kinase (PI3K) were mainly expressed in ovarian oocytes and granulosa cells [61]. The proliferation of luteinized-granulosa cells was significantly stimulated following 5 ng/mL Se treatment [61]. This proliferation, may be ascribed to the increased expression of biomarkers such as PCNA, cyclin-dependent kinase 1 (CDK1), phosphorylated adenosine monophosphate-activated protein kinase (p-AMPK; Thr172), and phosphorylated Akt (p-Akt; Ser473) and reductions in expression of p21 [61]. Similarly, the estradiol production was significantly enhanced [61], and the expression of antioxidant (*GPX* and *SOD2*) and steroidogenesis-related genes (*3β-HSD* and *StAR)* were also significantly increased following Se treatment [61]. Furthermore, the bioaccumulation of Se in bovine ovarian tissue has also been investigated recently [8]. Using X-ray fluorescence imaging, Ceko and colleagues have reported that Se was consistently identified in the granulosa cell stratum of large healthy follicles, meanwhile, compared to corpora lutea, approximately a ten-fold higher Se was observed in the wall of the bovine follicles [8]. Besides, among selenoprotein genes (*GPX1*, *GPX3*, *VIMP* and *SELENOM*) in bovine granulosa cells, only *GPX1* was significantly up-regulated in large healthy follicles compared to small healthy or atretic follicles [8]. These observations also suggest a potential role for this selenoprotein (antioxidant) in follicle dominance; i.e., shielding the dominant follicle from rising levels of ROS and resultant oxidative damage.

### 4.2. Evidence from Human Models Studies

The relationship between female fertility, Se status, and Se-dependent GPX (catalytic) activity has been demonstrated in very few studies that, in general, observed relatively low serum and follicular fluid levels to be linked with a higher infertility occurrence [82]. In 1995, Paszkowski and co-workers for the first time reported that there are noticeable traces of Se in human follicular fluid and manifestation of Se-dependent enzymatic activity [10]. One hundred thirty-five samples of follicular fluid obtained from 112 patients during transvaginal oocyte retrieval were evaluated and authors observed that the patients with idiopathic infertility had significantly reduced follicular Se levels compared to those with known causes of infertility such as tubal infertility or male factor related infertility [10]. Based on their findings, authors inferred that the antioxidant activity of selenoenzyme GPX in the microenvironment of follicles may play a significant role in gametogenesis and fertilization [10]. Recently, Ceko’s group investigated and proposed a substantial function and essentiality of GPX1 in determining the follicle growth, maturation, and dominance in human follicles [8]. For this purpose, human cumulus cells (CCs) derived from cumulus-oocyte complexes (COCs) obtained for both in vitro fertilization (IVF) and intra-cytoplasmic sperm injection were utilized [8]. These CCs recovered from COCs before embryo transfers that ensued in a pregnancy demonstrated significantly higher GPX1 expression compared to those that failed to result in a pregnancy [8]. The fact that many researchers have demonstrated a pronounced reduction in GPX1 expression under deficient dietary Se conditions highlights the possibility that ovarian pathologies may, partially, be ameliorated through Se supplementation [8]. These findings will serve as bases for epidemiological studies tending to outline the dietary uptake of Se, GPX1 expression in vivo, and the occurrence of ovulation related diseases [8,9].

## 5. Implication of Se in Ovarian Pathologies and Assisted Reproductive Technologies-related Oxidative Stress

The association between oxidative stress, reduced female fertility, and Se inadequacy is another scientific domain that is compelling us for further detailed investigations and understanding [83]. A possible instructive fact is that, excessive generation of ROS in endometriosis, such as in polycystic ovary syndrome, may presumably cause an extravagant expenditure of Se, because of its antioxidant attributes. However, then again, an elemental low Se status leads to an imbalance of antioxidant with an accretion of ROS which, by and large, is regarded as a predisposing factor for polycystic ovary syndrome. In any case, a low Se status in body causes a reduced resistance to free radical-induced injury; this may be attributed to its involvement in the formation of selenoproteins combined with its antioxidant ability [84].

### 5.1. Human Studies

The identification of patients suffering infertility or primary ovarian insufficiency (POI) with ovarian autoimmunity may lend the substitute treatment schemes from those due to chemotherapeutic, environmental, genetical, and radiation related factors [85]. Selenium-binding protein 1 (SBP1) is unique to ovarian autoimmunity related with infertility and POI [85]. Edassery and colleagues identified antigens frequently affiliated with serum auto-antibodies in women with idiopathic infertility and POI. Significantly higher levels of SBP1 were observed in women suffering from idiopathic infertility and premature ovarian failure [85]. In India, Singh et al. studied the markers of oxidative stress in the follicular fluid of women with endometriosis and tubal infertility undergoing IVF and observed a significantly (compared to controls) increased production of ROS and malonaldehyde in the follicular fluid of patients suffering from endometriosis [86]. Moreover, significantly lower Se levels were recorded in infertile women with endometriosis compared to women enduring tubal infertility [86]. In 2013, a study from Turkey, demonstrated significantly lower plasma Se concentrations in 36 women with polycystic ovary syndrome compared to the control group (*n* = 33 body mass index-matched healthy women) [87]. These reduced Se levels in women with polycystic ovary syndrome may be associated to hyperandrogenism [87].

Furthermore, it has been reported that appropriate supplementation of micronutrients including Se may have significant implication in the redox balance of follicular micro-environment and, consequently, on IVF results [88]. Two recent ART-related clinical studies (Luddi et al. [88] and Jiménez Tuñón et al. [89]) have demonstrated that Se supplementation (in the form of micronutrient supplement) may ameliorate the outcome of IVF (Table 3). Both of these studies were relatively underpowered, with very small number of enrolled subjects in either case. Another apparent limitation of these two studies is that Se was supplemented in combination with other micronutrients, therefore, the camouflaged effects of other essential trace elements cannot be entirely ruled out. These findings, to some degree, may serve as a useful foundation for the clinical practitioners in the management of women undergoing IVF treatment [88]. Nevertheless, we may benefit more by extending our apprehension of maternal nutrition in pregnancy to incorporate simultaneous mechanistic and epidemiological studies, particularly on multiple micronutrients and their interactions in order to fully elucidate the importance of these factors in successful reproduction and pregnancy [90].

### 5.2. In Vitro Studies

Generally, the embryos are, in particular, sensitive to oxidative damage, since they reportedly have lower antioxidant enzymatic activity compared to adults [91,92]. Nevertheless, in order to maintain the viability and quality of embryo, a rigorously characterized culture media is a prerequisite. Albeit, many embryo culture media are available nowadays, the optimum milieu is not greatly achieved and investigations focusing on blastocyst-promoting substances are still en route [93,94]. To ensure the adequate biosynthesis of selenoprotein in cultured cells, Se is contained in all mainstream tissue culture media [94,95,96]. For its potential enzymic activity, Se-dependent GPX does not only require Se and pyridoxine but, it also depends on glutathione (GSH) availability [97]. The amount of GSH in the porcine oocyte surmounts the needs for fertilization, but during pre-implantation development, embryonic GSH unremittingly reduces and attains a lowest point at the blastocyst stage. It is noteworthy that, the highest levels of endogenous ROS are also generated at this stage [98]. Presumably, these lower and higher levels of GSH and ROS respectively, have a possible function in the process of normal differentiation [99,100]; therein, ROS may have an essential functional and regulatory role pertaining to the apoptosis in the mouse blastocyst [99].

Selenium supplementation in embryo culture has been implicated in improving the developmental competence and overall quality of porcine parthenogenetic embryos by limiting the oxidative injury and programmed cell death [94]. The addition of sodium selenite (2.5 and 25 ng/mL) to the culture medium enhanced the blastocyst rate, cell number, and inner cell mass rate, reduced the apoptosis, the ratio of *BAX/BCL-xL* genes and the expression of Caspase 3, and improved the expression of GPX and ERK1/2 in porcine parthenogenetic embryos [94]. Moreover, Se supplementation has also been implicated in improving the intracellular GPX concentrations and activity, modulating the gene expression during in vitro oocyte maturation and ameliorating the blastocyst development and quality of in vitro fertilized bovine embryos. Recently in China, one research group has studied the Se concentrations in follicular fluid and the effects of Se supplementation on meiosis, DNA integrity in CCs, developmental competence of oocytes, GPX activity in denuded oocytes, and expression of Se-related genes in the yak oocytes during in vitro maturation [53]. Following supplementation of in vitro maturation medium with Se (2 and 4 µg/mL sodium selenite), the DNA damage was significantly reduced in cumulus cells. Meanwhile, total GPX activity, blastocyst formation rate, and the expression of selenoprotein-related genes were significantly increased in Se supplemented groups compared to the control (0 µg /mL sodium selenite) group [53].

In another in vitro study elucidating the role of sodium selenite, Abedelahi’s group reported that Se supplementation (5 or 10 ng/mL sodium selenite added in culture medium) ameliorated the ROS-induced oxidative stress and improved the total antioxidant capacity (TAC) and GPX activity in pre-antral follicles obtained from vitrified and non-vitrified ovarian tissues [101]. The development rate of follicles, oocytes, and embryos was significantly improved in Se treated groups [101]. Similarly, in their earlier in vitro study, the same group demonstrated that sodium selenite (5 and 10 ng/mL sodium selenite added in culture medium) exerts a dose-dependent ameliorative effect on growth and viability of follicles and oocytes, and these effects are presumably due to its proliferative and broader antioxidant capacity [102]. Taken as a whole, these findings, in some way, represent an approach towards attaining improved in vitro oocyte maturation and developmental capacity in mammalian oocytes and embryos, and may serve as a base for future research in this domain.

## 6. Effects of Maternal Dietary Se Supplementation

During gestation, dietary Se supplementation cannot only enhance antioxidant activity and stimulate the production of estradiol, progesterone and T_4_, but it also improves the overall metabolism of major nutrients [103]. In a few recent studies, the effects of maternal supplementation with organic and inorganic Se in combination with other vitamins such as pyridoxine were evaluated. It has been reported that pyridoxine has an important implication in the adequate flow of organic Se towards the Se-dependent GPX system in response to oxidative stress [104,105,106]. In any case, animal studies reporting the effects of maternal dietary Se supplementation on oxidative stress, antioxidant activity, embryo development, and reproductive efficiency are presented in Table 4.

## 7. Selenium and Placental Oxidative Stress

Selenium supplementation is considered an essential tool for the expressions and activities of endogenic antioxidants such as GPX and TXNRD; thus, it may likely ameliorate the oxidative stress- induced damage in experimental cell models, furnishing key mechanistic insights related to the safer utility of trace mineral supplementation in alleviating certain gestation-related diseases [32,110,111,112]. Placental oxidative stress is an important element in the pathophysiology and development of pregnancy-related complications; i.e., pre-eclampsia, intra-uterine growth restriction, gestational diabetes, and preterm labor [110]. A thorough apprehension of placental oxidative stress-induced trophoblastic apoptosis might furnish insights regarding the possible novel interventions for pre-eclampsia, which reportedly accounts for over eighty thousand premature births each year in the USA and, with the same token, is reckoned to cause around seventy-five thousands maternal deaths each year world-wide [19,111]. Recently, few reports have highlighted the possible mechanisms through which Se improves the overall mitochondrial function of trophoblastic cells and triggers mitochondrial biogenesis [32,59,110,111,112]. The findings of these studies have demonstrated that the ameliorative effects of Se are mediated through improved antioxidant generation and function, and a reduction in ROS production therefore escorting the mitochondrial function as well as increased expression of biogenesis activators [112]. Besides, SELENOH has been reported to activate the transcription factors such as NRF1 and PGC-1α to ameliorate mitochondrial biogenesis in trophoblastic cells [113]. These findings are enticing and will be useful for understanding the putative mechanisms through which trophoblastic cells react and respond to oxidative stress and how Se regulates the upregulation of associated genes and ameliorates the cell survival rate and their invasive ability by mediating the mitochondrial activity [114]. Recent studies assessing the role of Se supplementation in ameliorating the mitochondrial biogenesis and function in placental trophoblastic cell lines are detailed in Table 5.

## 8. Role of Se in Pregnancy

It is well established that Se participates in the bio-synthesis of selenoproteins such as GPX, SELENOP, TXNRD family, and may have an essential role in pregnancy. In particular, oxidative injury may be increased during this phase [115]. Consequently, antioxidant defense mechanisms have a central role to play in modulating events mediated by oxidative stress and could be related to perinatal morbidity and mortality [116,117,118]. It must be emphasized that the implication of Se status in the progression of pregnancy complications is yet to be properly instituted. Nevertheless, the association between low Se status and the occurrence of adverse fetal outcomes has been suggested. Because a larger number of available studies are either cross-sectional or case–control, their findings are not suitable to the assessment of causal relationships. Therefore, it could be assumed that either low Se status is implicated in the causal process of such pregnancy complications and subsequent outcomes or simply indicate a maternal physiological response to the raised oxidative stress states [119].

The hypothesis, “whether the pre-pregnancy Se status is of more significance in precluding the hypertensive pregnancy conditions than status in pregnancy”, itself is still inconclusive and needs further elucidation [15]. The periconceptual events that are potentially to be affected by Se status include the oocyte development, fertilization, and implantation [8,15]. Nevertheless, the recommendations prevail for increased Se intake during the gestation period [120]. Moreover, the best timing of Se supplementation during pregnancy is one of the well identified gaps amongst the studies concerning the role of Se on reproductive health; therefore, hitherto it is currently unclear what exactly should be the optimum timing of Se supplementation [121]. The scarcity of such key information pertaining to Se is by and large endorsed by other reports encouraging further elucidation with regard to the potential impact of appropriate timings of trace element supplementation on pregnancy results [122,123]. In 2017, Mamon and Ramos conducted a study to figure out the optimal timing of Se supplementation (3.0 μg per day) during pregnancy at different stages of the peri-conception period in a murine model [121]. Improved quality of blastocysts and reduced pre-implantation loss was observed in pre-gestation only and gestation only supplementation groups compared to the control and pre-gestation-to-gestation supplementation groups [121]. These findings highlight that pre-gestation only and gestation only are the optimal peri-conception phases for Se supplementation to achieve improved developmental competence in blastocysts and pre-implantation success [121].

**Figure 1:**
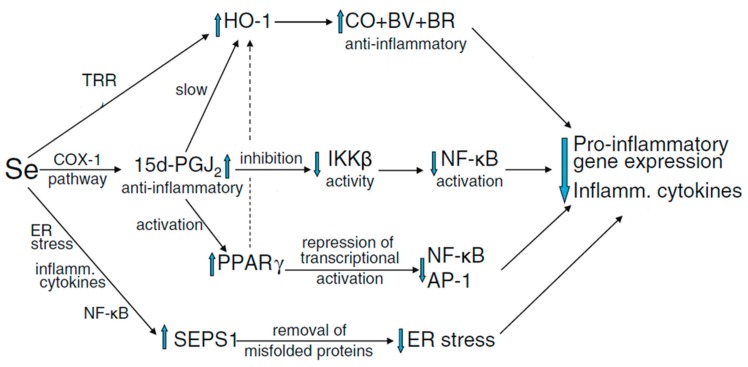
Possible mechanisms by which Se may reduce inflammation resulting from oxidative and ER stress. Abbreviations: ER, endoplasmic reticulum; SEPS1, selenoprotein S; 15d-PGJ2, 15-deoxy-∆12,14-prostaglandin J2; IKKβ, IκB-kinase β; TRR, thioredoxin reductase; BV, billiverdin; BR, billirubin; PPAR-γ, peroxisome proliferator-activated nuclear receptor-γ (adopted by permission from [29], Springer Nature, New York, USA).

Recently, a very small number of properly powered and well-conducted studies have shown that Se is implicated in a number of complications related to pregnancy [15,37,124,125]. Positive correlation of serum Se and SELENOP levels is generally observed in populations with a borderline Se supply [124]. Furthermore, it has been shown that during the course of normal pregnancy, the whole blood Se concentrations fall considerably (12%) with advancing gestation [37,126,127]. Keeping the plasma volume expansion aside, another potential reason of a fall in concentration of Se in blood during pregnancy is probably to be the transport of Se to the fetus mediated via SELENOP, which is expressed in the placental tissues [68,72]. Conversely, higher Se status has been linked with a lower risk of miscarriage and preterm birth, however, the level of manifestation from randomized controlled trials is stronger for the beneficial role of higher Se intake and or status in pre-eclampsia and autoimmune thyroid disease. The capability of Se to ameliorate the oxidative stress, endoplasmic reticulum stress and inflammation (Figure 1), to shield the endothelium, to regulate the production of eicosanoid, to modulate the vascular tone, and repress the infection is probably crucial in the context of these conditions [29].

In one UK based high-quality double-blind, placebo-controlled, pilot trial, two hundred thirty primiparous pregnant females were randomized to Se treatment (60 μg/d, as Se-enriched yeast) or placebo from weeks twelve to fourteen of pregnancy till delivery [37]. Between 12 and 35 weeks, the whole blood Se concentrations were significantly increased in the Se supplemented group but reduced significantly in the placebo block. Similarly, at 35 weeks, significantly higher levels of whole blood Se and plasma SELENOP were recorded in the Se supplemented group compared to the placebo block [37]. Moreover, an inverse relationship between Se supplementation and serum soluble vascular endothelial growth factor receptor-1 (sFlt-1) was demonstrated in this study, suggesting that Se supplementation was beneficial in limiting the production of sFlt-1 that has anti-angiogenic effects and contributes to the pathophysiology of pre-eclampsia. This is the only report available to have investigated the effect of Se supplementation in a population with low Se status that studied an outcome related to the risk of pre-eclampsia [37]. In any case, there has been another recent report on Se status declining in Polish pregnant women trimester by trimester, causing the majority of pregnant women to develop severe Se deficiency. A progressive decline in the mean levels of Se and SELENOP were observed during the course of gestation in enrolled pregnant women [124]. In this study, a positive correlation was observed between serum Se and SELENOP concentrations across all three trimesters in the full group of pregnant women [124]. Similar observations were recorded in another recently reported Polish Mother and Child Cohort in which plasma Se concentrations declined from 1st to 3rd trimester by 23 % (48.3 +/− 10.6 to 37.3 +/− 9.8 μg/L) [125].

Intra-uterine growth restriction, (affecting 10–15 % of pregnant women) is a principal cause of peri-natal morbidity and mortality [128,129]. It has been reported that higher levels of ROS and inflammatory biomarkers have a central play in development and pathology of intra-uterine growth restriction [130,131]. Mesdaghinia and colleagues conducted a randomized double-blind placebo-controlled trial to evaluate the effects of supplemental Se on clinical signs and metabolic status in pregnant women (*n* = 60) at risk of bearing the intra-uterine growth restriction [131]. Entrants were randomly designated to take either 100 μg Se (tablet) supplement (*n* = 30) or placebo (*n* = 30) for full ten weeks (from seventeen to twenty-seven weeks of pregnancy). Following ten weeks long Se supplementation, a higher number of females in the Se-treated block showed higher pulsatility index (<1.45) in comparison to the placebo block [131]. Moreover, the plasma levels of TAC, GSH and high-sensitivity C-reactive protein were also significantly changed in the Se-treated block compared to the placebo block [131].

It is pertinent to mention that Se has also been implicated in other pregnancy associated complications such as recurrent miscarriage [56,132,133], small for gestational age infants [119,134], and postpartum thyroid dysfunction [135]. There is evidence from some randomized controlled trials and observational studies that Se (probably as component of selenoproteins) could reduce thyroid peroxidase antibodies levels, hypothyroidism, and postpartum thyroid diseases [136]. Pregnant women positive for thyroid peroxidase antibodies are at a higher risk of developing postpartum thyroid dysfunction (PPTD) and permanent hypothyroidism [135]. Selenium has also been implicated in reducing thyroid inflammatory activities in subjects with autoimmune thyroiditis [135]. In one high-quality randomized, controlled trial conducted in thyroid peroxidase antibodies-positive (TPOAb-positive) pregnant women [135], it was reported that Se supplementation (200 μg selenomethionine/day) significantly reduced TPOAb titer, thyroiditis, postpartum thyroid disease, and permanent hypothyroidism compared to the placebo group [135]. Even though the ingratiatory manifestations are already available in extant literature suggesting that supplemental Se would, in some way, benefit some of these complications in Se-deficient subjects; nonetheless, the results from ongoing (if any) or future intervention trials have the potential to reinforce or controvert the argument regarding increasing Se uptake.

## 9. Implication of Se and Selenoproteins in Reproductive Cancer

The implication of Se in carcinogenesis and cancer therapy has been a topic of controversial discussion for many decades, and the prevention, inhibition, or progression of cancer by selenoproteins is still a subject of on-going debate [137]. Ovarian cancer is ranked as the 5th leading cause of cancer deaths in women and has the highest overall mortality and a poor five-year survival rates [138]. Over ninety percent of the cases of ovarian cancer are epithelial ovarian cancer (EOC), which presents a series of etiologically and molecularly distinct disorders [138]. Some evidence exists in the available literature regarding the implication of Se and selenoproteins in female reproductive cancers such as ovarian cancer [139]. Significantly lower mean concentrations of Se and GPX activity have been noticed in the plasma of subjects bearing benign neoplasia and cancer of the uterine cervix, uterine corpus or ovary compared to healthy women [140]. These findings suggest that the lower antioxidant capacity (Se-related) may favor the development of female reproductive system-related tumorigenic diseases [140]. Agnani et al. reported a stage-dependent decrease in the activity of GPX3 in women suffering papillary serous ovarian cancer [139]. In the meantime, reduced serum GPX3 levels are observed in women with recurrent disease and these level-dependent reductions become more marked when patients and controls are stratified to admit only women older than 50 years of age [139]. Furthermore, both in rats as well as humans, *GPX3* was also observed to be down-regulated in all grades of endometrial adenocarcinoma, irrespective of tumor grade [139,141]. In some of the previous studies, it has been reported that expression of GPX3 is higher in clear cell epithelial ovarian carcinoma tissues compared to the control [142,143,144]. Even though the underlying molecular mechanisms are not yet sufficiently understood, these results indicate that GPX3 activity is tumor-specific [138]. In one recent report, Wu and colleagues have reported that GPX3 was observed to be down-regulated in a platinum-sensitive group compared to the platinum-resistant group [138]. The putative mechanisms involved in mediating the GPX3 tumor-suppressing role are primarily attributed to promoter hyper-methylation, dysregulation of c-Met expressions, and the enzymic role of antioxidants that scavenge the detrimental free radicals [138]. However, the exact underlying mechanisms, in response to antitumor drugs, remain largely elusive and require further elucidation [138]. Nevertheless, Se and selenoproteins may provide targets for reproductive cancer treatment or prevention strategies.

## 10. Conclusions

In this review, we provide a comprehensive spotlight on the potential role of Se and selenoproteins in overall reproduction efficiency and many disorders related to both animal and human reproduction. It is obvious from the findings of previous studies (both animal and human) that Se and selenoproteins are essential for optimal reproduction in females, presumably because of their implication in the regulation and modulation of antioxidant balance.

The role of Se and selenoproteins in many domains of female reproduction, such as the effects of maternal dietary Se supplementation on oxidative stress, role of Se in fetal ovarian development and function, and the implication of Se supplementation in ameliorating the placental oxidative stress, is poorly understood and requires through consideration. Moreover, little attention has been paid to the pre-conception period, or focus on general reproductive efficiency and health when it comes to ovarian physiology, in particular, the synthesis of hormones and the development of ovarian follicles. Only recently has some evidence on the possible role of Se and selenoproteins in oocyte maturation and fertilization emerged from in vitro animal trials; but large, well powered, in vivo controlled trials are still sparse. Furthermore, the level of evidence regarding the role of Se and selenoproteins in different ovarian pathologies including cancer is very low and inconclusive. In the meantime, the implication of Se and selenoproteins in ameliorating the oxidative and nitrosative stress induced in assisted reproductive technologies such as IVF and embryo development is not yet sufficiently understood. Besides, there are many other questions regarding the role of Se and selenoproteins in female reproduction (animal and human) that remain unrequited. Answers to these questions as well as a further elucidation of the roles of Se and selenoproteins should help to explicate the potential biological functions of this potent micronutrient (Se) in female reproductive efficiency and health.

The response curve for Se supplementation appears to be U-shaped; i.e., beneficial in Se-deficient individuals and with potential health related risks in those who are supplemented in excess concentrations [145]. Therefore, we must be careful not to encourage the excessive intake of Se supplements, in particular people of adequate or high status. As for the implication of Se supplementation in pregnancy, the outcomes of sparse yet high-quality properly powered, randomized, controlled trials in populations of relatively low Se status are enticing [37], however, higher numbers of such trials (double-blind, placebo-controlled, two-group studies with proper follow-up) are required to further elucidate the exact underlying molecular and biochemical mechanisms through which Se or selenoproteins might have beneficial effects in reducing the markers of risk of pregnancy-related complications such as pre-eclampsia. In this context, the current ongoing trials (if any) and the meta-analysis should soon enable the development of proper recommendations. Nevertheless, as a matter of fact, there is a profuseness of specialized supplements (containing Se) marketed to pregnant women and the percentage of pregnant women consuming such supplements is now very high in developed countries; it is getting increasingly hard to enroll subjects (pregnant women) in randomized control trials.

To sum up, the level and quality of available evidence regarding the ameliorative effects of both Se and selenoproteins in female reproduction are high; however, the volume of such research is not yet sufficient enough to draw solid conclusions. Therefore, studies elucidating the potential effect of Se supplementation and selenoproteins (i.e., GPX, SELENOP, SELENOS and allied) in ovarian function, pregnancy-related complications, and overall female reproductive efficiency would be of great value and will add more to the tally of the already known benefits of this trace mineral.

## Figures and Tables

**Table 1:** Brief description of select mammalian selenoproteins having implication/role in fertility, reproduction, and development.

Selenoprotein	Symbol [5]	General Description/Function [1,6,7]	Potential Implication in Reproductive Function
Glutathione peroxidase 1	GPX1	Antioxidant protection	Important role in female reproductive function; i.e., implication in determining the follicle growth, maturation, and dominance in both cows and women [8,9]. Implication in the follicular microenvironment [10]. Potential antioxidant role in follicle dominance protecting the dominant follicle from increasing levels of reactive oxygen species (ROS) [8,9].
Glutathione peroxidase 2	GPX2	Antioxidant protection	Implicated in protection of embryos and extra-embryonic tissues against ROS generated in ontogenetic periods [11].
Glutathione peroxidase 3	GPX3	Maintenance of cellular redox status Antioxidant in extracellular fluids	Gpx3 has been identified as an essential enzyme in the defense against oxidative stress during the postovulatory process of endometrial remodeling (decasualization) in preparation for implantation by reducing H_2_O_2_ in the endometrium [12]. Implicated in normal human pregnancy and birth [13]. Implicated in maternal-fetal selenium transfer mechanism [14]. Role in pre-eclampsia and pregnancy-related hypertensive conditions [15]. Implicated in preventing the oxidative stress- induced cell apoptosis during growth of large healthy follicles [16].
Glutathione peroxidase 4	GPX4	Detoxification of lipid hydroperoxides, Antioxidant role in membranes, serves as structural protein in sperm, Apoptosis. Membrane-associated. Present at high concentrations in the testis, where it is important for sperm motility and viability, male fertility	Essential for embryonic development [17,18]. Potential implication in pre-eclampsia [19,20,21]. Implication in male fertility [22].
Glutathione peroxidase 5	GPX5	Antioxidant role during sperm maturation, H_2_O_2_ scavenger [23]	Implicated in protection of sperm from oxidative damage that could compromise their integrity and, as a consequence, embryo viability [24].
Thioredoxin reductase 1	TXNRD1	Part of the thioredoxin system, Antioxidant role, redox regulation, cell signaling. Controls activity of transcription factors, cell proliferation, apoptosis	Implication in early embryonic development [25,26].
Thioredoxin reductase 2	TXNRD2	Part of the thioredoxin system, Antioxidant role, redox regulation, cell signaling	Potential role in embryogenesis [27].
Thioredoxin-glutathione reductase	TXNRD3	Part of the thioredoxin system, Antioxidant role, redox regulation, cell signaling	Role in disulfide bond formation and sperm maturation [28].
Iodothyronine deiodinase 1	DIO1	Conversion of T_4_ to T_3_ and T_4_ to reverse T_3_, Production of T_3_ in the thyroid and peripheral tissues	Implication in autoimmune thyroid disease and postpartum thyroid disease [29]. Dio2 and Dio3 are highly expressed at the site of implantation in pregnant rat [30]. High expression of DIO3 at utero-plecental unit and fetal epithelia has also been reported in human, Potential role in preventing the overexposure of fetus to T_3_ [31].
Iodothyronine deiodinase 2	DIO2	Conversion of T_4_ to T_3_, T3 production in peripheral tissues	
Iodothyronine deiodinase 3	DIO3	Conversion T_4_ to reverse T_3_, Production of rT_3_	
Selenoprotein H	SELENOH	Not fully known, potential implication in upregulation of genes relevant to glutathione synthesis	Implicated in placental oxidative stress by regulating mitochondrial biogenesis in trophoblasts (Swan-71, JEG-3 and BeWo cells) [32].
Selenoprotein P	SELENOP	By and large implicated in Se transportation and antioxidant defense, Regarded as a major contributor to plasma selenium and a good indicator of Se status, Essential for male fertility; its deficiency leads to infertility characterized by abnormal spermatozoa in mice	Implicated in maternal-fetal selenium transfer mechanism [14]. Implication in male fertility [33,34]. Involved in delivery of Se to spermatogenic cells [35]. Required for mouse sperm development [36]. Potential implication in pregnancy and pre-eclampsia [13,15,37].
Selenoprotein S	SELENOS	Cellular redox balance, Possible influence in inflammatory response	Relevant to pre-eclampsia [29,38]. Localized to the granulosa cells of large healthy and atretic follicles and also expressed in the thecal layers. Potential role in bovine follicular development [8].
Selenoprotein V	SELENOV	Unknown, possible role in redox regulation	Testis-specific expression in rodents [39], in situ hybridization experiments have shown high levels of Selenov mRNA in seminiferous tubules in mice, but its exact role in spermatogenesis remains elusive [3,39].

**Table 2:** Recommended Dietary Allowance (RDA) of selenium for human and different animal species.

Species	RDA
Human	Male: 55 µg/day * [43]; Female: 55 µg/day * [43] Female during pregnancy: 60 µg/day * [43]
Sheep	100–200 µg/kg DM of feed/day [44]
Goat	100–200 µg/kg DM of feed/day [44]
Growing Pigs Gestating and Lactating Sows	150–300 µg/kg DM of feed/day [45] 150 µg/kg DM of feed/day [45]
Horse	100 µg/kg DM of feed/day [46]
Donkey	150 µg/100 kg body weight [47]
Dairy cows	100 µg/kg DM of feed/day [48]
Beef cows	300 µg/kg DM of feed/day [49]
Calves	100 µg/kg DM of feed/day [48]
Camel	400–800 µg/day [50]

* Healthy adults (age: >18 years); values for United States and Canada. For further detailed information regarding RDAs of Se; readers are referred to Rayman et al. [51]. DM, Dry Matter.

**Table 3:** Studies demonstrating the implication of selenium (as micronutrient supplement) in ameliorating the in vitro fertilization (IVF) outcomes.

Study Design	Type and Duration of Treatment	No. of Subjects Enrolled	Key Results Relevant to IVF	Ref.
Open label preliminary clinical trial (Italy)	Elevit, Bayer containing Se (50 μg) for three months	18 patients aging >39 years; undergoing IVF/intracytoplasmic sperm injection (ICSI) treatment	The follicular fluid and serum proteins were protected from oxidative injury when aged women were supplemented with micronutrients ahead of IVF cycles. Significant increase in the mean values of good quality oocytes (recovered from women following the micronutrient supplement) was also observed.	[88]
Double-blind, randomized prospective study (Spain)	Seidivid, containing Se (27.5 μg) 2 months prior to ovarian puncture	120 patients going through assisted reproductive interventions	Significantly improved embryonic quality was recorded. No significant improvement in the results (relating to follicle number, oocyte maturation, quality of vitrified embryos) was recorded.	[89]

**Table 4:** Effects of maternal dietary Se supplementation on oxidative stress, antioxidant activity, embryo development and reproductive efficiency.

Model	Treatment Regime	Key Findings as Reported by Authors	Ref.
Pregnant goats	0 mg (control group), 0.5, 2.0 and 4.0 mg Se/kg DM during gestation period	Organic Se supplementation significantly improved the total antioxidant capacity (TAC), activity of GPX and SOD. The levels of estradiol, progesterone and T_4_ were also significantly increased	[103]
Gilts	Organic (0.3 mg/kg Se- enriched yeast) and inorganic (0.3 mg/kg sodium selenite) Se (combined with pyridoxine)	Organic Se (with pyridoxine) substantially induced the transcriptome of porcine expanded blastocysts compared to gilts in the control group. Organic Se (with pyridoxine) more noticeably affected the metabolism in porcine expanded blastocysts compared to those supplemented with inorganic Se (with pyridoxine). Organic Se selectively stimulated the genes implicated in antioxidant defense. In expanded blastocysts from organic Se supplemented gilts, other members of the thioredoxin family and ubiquinones seem to embellish the antioxidant activity and the modulation of cell proliferation	[104]
Gilts	Basal diet 0.3 mg/kg and 2.4 mg/kg of Se and pyridoxine, respectively; 0.3 mg/kg sodium selenite without pyridoxine; 0.3 mg/kg sodium selenite +10 mg/kg pyridoxine; 0.3 mg/kg of Se-enriched yeast without pyridoxine; 0.3 mg/kg of Se-enriched yeast +10 mg/kg pyridoxine	Both Se level and source significantly enhanced Se concentration in the organs of gilts and the embryos. Selenium level showed implication on total GPX activities in mitochondria	[105]
Hyperovulatory first-parity gilts	Control group: Se 0.2 mg/kg; control +sodium selenite 0.3 mg/kg; control +Se-enriched yeast 0.3 mg/kg	The Se content of individual embryos was higher in the Se-supplemented group compared to the control. The uterine transfer of Se to embryos was ameliorated and this was concomitant with an improved embryo development. GPX was improved following Se supplementation	[107]
Multi-parous sows	sodium selenite 0.30 mg Se/kg organic Se 0.30 mg Se/kg	Organic Se significantly increased SOD activity, GPX and glutathione (GSH) content, Se level, total antioxidant activity, number of live and weaned pigs. Whereas organic Se significantly decreased malondialdehyde (MDA) contents compared to the inorganic Se	[108]
Rat	Se deficient (0.01 ppm) Se sufficient (0.5 ppm)	In Se deficient group, higher mortality at birth; reduced viability and survival parameters, impaired growth, and development of liver in offspring accompanied with reduced hepatic Se concentrations, GPX and CAT activities, and increased SOD activity and protein oxidation were observed. However, Se sufficient diet ameliorated all aforementioned indices.	[109]
Gilts	Basal diet (control group); Basal diet +0.3 mg/kg of sodium selenite; sodium selenite +10 mg/kg pyridoxine; Basal diet +0.3 mg/kg of Se-enriched yeast; Se-enriched yeast +10 mg/kg pyridoxine	Organic Se supplementation along with dietary B_6_ (pyridoxine) increased the expressions of *GPX1*, *GPX3*, *GPX4*	[106]

**Table 5:** Main characteristics and results of recent studies assessing the effect of selenium supplementation on mitochondrial function and expression of mitochondrial biogenesis markers in placental trophoblast cell lines.

Model	Treatment Regime	Key Findings and Implications	References
Placental trophoblast-like cell lines (BeWo, JEG-3 and Swan-71)	Sodium selenite (100 nM) for 24 h Selenomethionine (500 nM) for 24 h	Following Se supplementation, significantly higher mitochondrial respiration was observed in trophoblast-like cell lines compared to untreated control group. Interestingly, the treated Swan-71 cells were observed to have higher mitochondrial content (measured on basis of the ratio of mitochondrial DNA [mtDNA] to nuclear DNA [nDNA]) in both Se supplemented groups. The expression of four mitochondrial genes; i.e., *MTRT1, MTRT2, MTRT3* and *MTRT4* genes was significantly improved in cells treated with inorganic Se, whereas expression of only two out of these four genes was significantly improved in cells treated with organic Se. The other mitochondrial biogenesis markers; i.e., SELENOH, peroxisome proliferator-activated receptor γ coactivator-1alpha (PGC-1α) and nuclear respiratory factor-1 (NRF-1) were also significantly improved in Swan-71 cells after treatment with both organic and inorganic Se.	[110]
First trimester villous placental tissue	Sodium selenite (100 nM) for 4, 12, 14, 24, 48 or 96 h.	The mitochondrial respiration (increased oxidative phosphorylation through complex IV) was significantly improved in trimester villous placental tissue explants following four hours of culture in sodium selenate treated group.	[110]
Placental trophoblast cell lines (BeWo, JEG-3 and Swan-71)	Sodium selenite and Selenomethionine. (Dose-dependent manner i.e., 0, 50, 100, 200, 400, 800, 1200, 1600 or 2000 nM for 24 h). Cells were treated with mitochondrial inhibitors rotenone or antimycin to induce the oxidative stress	The expression and activity of key selenoproteins, namely, GPX and TXNRD was significantly improved (in a dose-dependent manner) in Swan-71 cells following supplementation with both sources of Se. Selenium supplementation significantly reduced the ROS- induced oxidative stress in both treated groups. Moreover, rotenone or antimycin-induced cellular toxicity was also significantly reduced in Swan-71 cells following Se supplementation. The uncontrolled apoptosis and expression of Bcl-2 in BeWo, JEG-3 and Swan-71 cells were significantly reduced.	[111]
Hypoxia exposed human extravillous trophoblast cell line HTR-8/Svneo	0.5 nM selenium for 72 h	Selenium supplementation under hypoxia increased the migration and proliferation of trophoblastic cells by ameliorating the mitochondrial oxidative stress and improved the expression of antioxidant genes i.e., heme oxygenase 1, 2 (*HO-1* and *HO-2*) and *SOD*.	[114]
Placental trophoblast cell lines BeWo, JEG-3 and Swan-71	Sodium selenite (100 nM)	Selenium supplementation efficaciously stimulated the mitochondrial biogenesis, increasing the number of mitochondria in trophoblastic cells. The consumption of oxygen was comparatively higher in the Se treated cells compared to the control group. The mitochondrial respiration indicated that the oxygen streaming was significantly enhanced in Se treated trophoblastic cells. The ratio of mitochondrial gene/nuclear gene was higher in the cells supplemented with Se. Meanwhile, Se supplementation also improved the expression of SELENOH, NRF-1 and PGC-1α	[112]
Trophoblast cells (BeWo, JEG-3 and Swan-71)	Sodium selenite (100 nM) Selenomethionine (500 nM) Oxidative stress was induced in the cells with the addition of increasing doses of Antimycin C, Rotenone and Resazurin	A dose-dependent reduction in the cellular activity in BeWo, JEG-3 and Swan-71 was observed when treated for 4 h with increasing concentrations of Antimycin (40–320 μM) and Rotenone (100–800 nM). However, prior incubation with sodium selenite and selenomethionine protected the trophoblast cells from oxidative stress. Following Se supplementation, the activity of GPX and TXNRD was significantly improved (in a dose-dependent manner). Induced cellular oxidative stress was significantly ameliorated following supplementation with both organic and inorganic Se.	[32]
Human trophoblast cell lines BeWo and JEG-3	Sodium selenite (100 nM) Selenomethionine (500 nM) Oxidative stress was induced using H_2_O_2_ (0–800 µM), tert-butyl hydrogen peroxide (t-butyl H_2_O_2_, 0–50 µM), cumene hydrogen peroxide (cumene H_2_O_2_, 0–50 µM)	The activity of GPX and TXNRD was significantly improved (in a dose-dependent manner). Induced cellular oxidative stress was significantly reversed following supplementation with both organic and inorganic Se.	[59]
